# Utilization of integrated angiography-CT interventional radiology suites at a tertiary cancer center

**DOI:** 10.1186/s12880-020-00515-x

**Published:** 2020-10-15

**Authors:** Joseph P. Erinjeri, Raphael Doustaly, Grégoire Avignon, Achiude Bendet, Elena N. Petre, Etay Ziv, Hooman Yarmohammadi, Stephen B. Solomon

**Affiliations:** 1 Interventional Radiology Service, Department of Radiology, Memorial Sloan Kettering Cancer Center, 1250 York Ave, Suite H112, New York, NY 10021 USA; 2 GE Healthcare, 283 Rue de la Minière, 78533 Buc, France; 3 Rabin Medical Center, Petach-Tiqwa, Israel

**Keywords:** Angiography-CT, Interventional radiology

## Abstract

**Background:**

Integrated Angiography-Computed Tomography (ACT) suites were initially designed in the 1990’s to perform complex procedures requiring high-resolution cross-sectional imaging and fluoroscopy. Since then, there have been technology developments and changes in patient management. The purpose of this study was to review the current usage patterns of a single center’s integrated ACT suites.

**Methods:**

All procedures performed in 2017 in 3 ACT suites (InterACT Discovery RT, GE Healthcare) at a tertiary cancer center were reviewed retrospectively. Usage was classified as: Standard, in which the patient underwent a single procedure using either fluoroscopy, CT, or ultrasound (US); Combined, in which the patient underwent a single procedure utilizing both fluoroscopy and CT; or Staged, in which the patient underwent 2 separate but successive procedures using fluoroscopy and CT individually. The most frequently performed Combined and Staged procedures were further reviewed to determine how the different modalities were used. The duration of the most common Staged procedures was compared to analogous procedures’ durations in single modality rooms over the period Jan 2016 to Sep 2019.

**Results:**

A total of 3591 procedures were performed on 2678 patients in the 3 ACT Suites. 80% of patients underwent a Standard procedure using fluoroscopy (38%), CT (32%) or US (10%) and accounted for 70% of the room occupation time. Fourteen and three percent of the patients underwent Combined or Staged procedures, occupying 19 and 5% of the room time, respectively. The remaining procedures were classified as both Combined and Staged, representing 3% of the patients and 6% of the room occupation time. The most common Combined procedures were drainages, hepatic arterial embolizations or radioembolizations, arterial, and biliary interventions. The most common Staged procedures were multiple drainages and hepatic arterial embolizations followed by biopsies or ablations. The room occupation time for liver tumor embolization and ablation was significantly shorter (*p* < 0.01) when performed in a Staged fashion versus the analogous procedures in single modality room.

**Conclusion:**

An integrated ACT system provides the capability to perform complex Combined or Staged procedures as well as scheduling flexibility by allowing any type of case to be performed in the IR suite.

## Background

The concept of an integrated Angiography – Computed Tomography (ACT) suite was developed in the 1990’s in Japan [1] as a system to improve the precision of interventional procedures by using both real-time imaging (fluoroscopy) and high quality axial imaging (CT), which angiographic systems were not capable of providing at the time. The system combines an angiography unit with a CT scanner that shares the same patient table. This setting allows for performing CT, conventional fluoroscopy and angiography without the need to move the patient, thus lowering the risk of dislocating the needle/catheter or breaking sterile conditions. Furthermore, the system provides the ability to perform multiple procedures requiring different imaging modalities on the same patient and to perform advanced percutaneous interventions combining multiple imaging modalities for complex cases.

The ACT system was originally introduced in the oncologic setting [2], mainly in hepatic procedures, both therapeutic and diagnostic, of primary and metastatic tumors [3–6]. Other usage reports of these systems include the treatment of intractable ascites [7], treatment of advanced maxillary sinus cancer, [8] and a percutaneous drainage of mediastinal abcess [9]. In recent years, similar combined room settings were also utilized in the neuro-interventional setting for stroke treatment [10]. Moreover, in 2006 the first MR/fluoroscopy operating room was introduced for neurosurgical procedures [11], following the pattern of combined modality settings.

However, there have been many developments and changes in the field since the original introduction of the ACT suite concept including the introduction of C-arm Cone Beam CT technology nearly 15 years ago [12] with rapid improvement in detector technology and rotation speeds ever since [13]. Furthermore, the field of Interventional Oncology has progressed significantly since the 1990’s with the introduction of new transarterial therapies such as radioembolization and drug eluting beads along with new percutaneous procedures such as microwave ablations, irreversible electroporation, and cryoablation. In recent years, the emergence of combined vascular and percutaneous approaches has allowed for more efficient treatment of more diseases in different organs than was common practice 25 years ago. Due to continued improvements, it remains unknown how ACT is currently utilized in Interventional Oncology and what is its potential benefit.

The purpose of this study is to expand on our initial experience [14] with 3 integrated Angiography-CT Interventional Radiology (IR) suites at a tertiary referral cancer center during the year of 2017, discuss the workflow and clinical benefits compared to typical IR settings, and offer general recommendations for future utilization.

## Methods

### Patients

This retrospective study was approved by our Institutional Review Board with a waiver of informed consent. For this type of study formal consent is not required. For ACT utilization analysis, we retrospectively reviewed all patients who underwent procedures in one of three integrated ACT suites from January 2017 to December 2017. We also looked at procedure time and room occupation time of procedures performed at our institution from January 2016 to September 2019, for comparison between standard angiography rooms and ACT rooms.

### ACT interventional radiology suite

We analyzed the procedures performed in 3 different integrated ACT rooms. Two of them were equipped with an Innova 4100 angiography unit combined with a LightSpeed CT (ACT/SmartGantry Option LS system, GE Healthcare), and one was equipped with an IGS 540 combined with a Discovery RT wide bore CT (INTERACT Discovery RT, GE Healthcare). The angiography table was shared with the CT unit, which was mounted on a rolling platform, and as opposed to in conventional CT systems, the gantry was moving instead of the table during a CT scan. The ACT system had a user interface that allowed users to quickly switch the system into either imaging modality mode, and each system was equipped with a dedicated CT fluoroscopy kit.

In order to maximize patient access and scan range, the table was usually fully extended during CT scans, and more specifically during CT-guided procedures, though there was no specific requirement regarding the table’s longitudinal position to perform a CT acquisition. The ACT system allowed users to acquire multiple CT scans using a single scout image even if the user had changed the active modality from CT to fluoroscopy during the case.

Ultrasound devices used during the procedures were both the Logiq E9 or Logic S7 (GE Healthcare) systems.

### ACT suite usage analysis

The term “procedure” was considered to be the occurrence of a patient entering the ACT suite. Each procedure could have 1, 2, or more interventions done on the patient with each intervention done under guidance of either imaging modality.

The procedures were divided into 3 categories based on the imaging modality used during each intervention:

1. Standard, in which the patient underwent a single procedure using either fluoroscopy, CT or Ultrasound (US)
2. Combined, in which the patient underwent a single procedure utilizing both fluoroscopy and CT
3. Staged, in which the patient underwent two separate but successive procedures using fluoroscopy and CT individually (Table 1).

**Table 1:** Procedures classification

1st/2nd intervention	none	CT	XA	US	CT+ XA
CT	Standard	Standard	Staged	Standard	Staged + Combined
XA	Standard	Staged	Standard	Standard	Staged + Combined
US	Standard	Standard	Standard	Standard	Combined
CT+ XA	Combined	Staged + Combined	Stage + Combined	Combined	Staged + Combined

Procedure categories based on the imaging modality combinations of the 1st intervention (1st column) and the 2nd one (1st row). If more than 2 interventions were performed, the same logic was applied. All procedures were classified regardless the use of ultrasound in addition to the other modalities as it’s common practice in interventional radiology and not relevant for the current study

If a procedure had one intervention utilizing only one modality (CT or fluoroscopy) and a second intervention utilizing both, the procedure would be categorized as both a Combined and Staged procedure. Procedures where US was used in combination with either CT or Fluoroscopy were referred to as simple procedures since they are common practice in the standard IR suite and not relevant for the current study. The importance of Combined and Staged procedures in the ACT suites procedure mix was evaluated by comparing the procedure types occurrences and room occupation time.

For each procedure performed in ACT suites between January 2017 and December 2017, the following data relevant for the procedure was obtained: procedure description, date, type, room occupation time, body part treated, and imaging modalities used during the procedure (CT, Fluoroscopy, US).

The most frequently performed Combined procedures were reviewed to determine how each modality was used and in which purpose.

### Most common staged ACT procedure versus equivalent single modality room procedures comparison

In a second retrospective analysis, the procedure type, duration and the room occupation time were surveyed for all Standard and Staged procedures performed in the ACT suites between January 2016 and September 2019. The most common Staged procedure performed in ACT suite was compared to the 2 equivalent single procedures in terms of procedure duration and room occupation time. Due to the type of data available, only the Staged procedures where the gold standard in interventional oncology practice is the use of angiography for one intervention and CT for the other intervention were selected.

### Statistical analysis

Usage of the different imaging modalities was evaluated using standard analytic tools, both in terms of use per procedure and time spent using each modality.

The room occupation time of staged procedures was compared to the cumulative room occupation time averages of equivalent single procedures using a one sample t-test. The comparison of procedure durations was evaluated in the same way.

## Results

### Room usage

From January 2017 to December 2017, 2678 patients underwent 4298 interventions as part of 3591 procedures in the 3 ACT suites. Eighty percent of patients underwent a Standard procedure. The rest of the patients underwent Combined (14%), Staged (3%) procedures or both (3%).

**Fig. 1:**
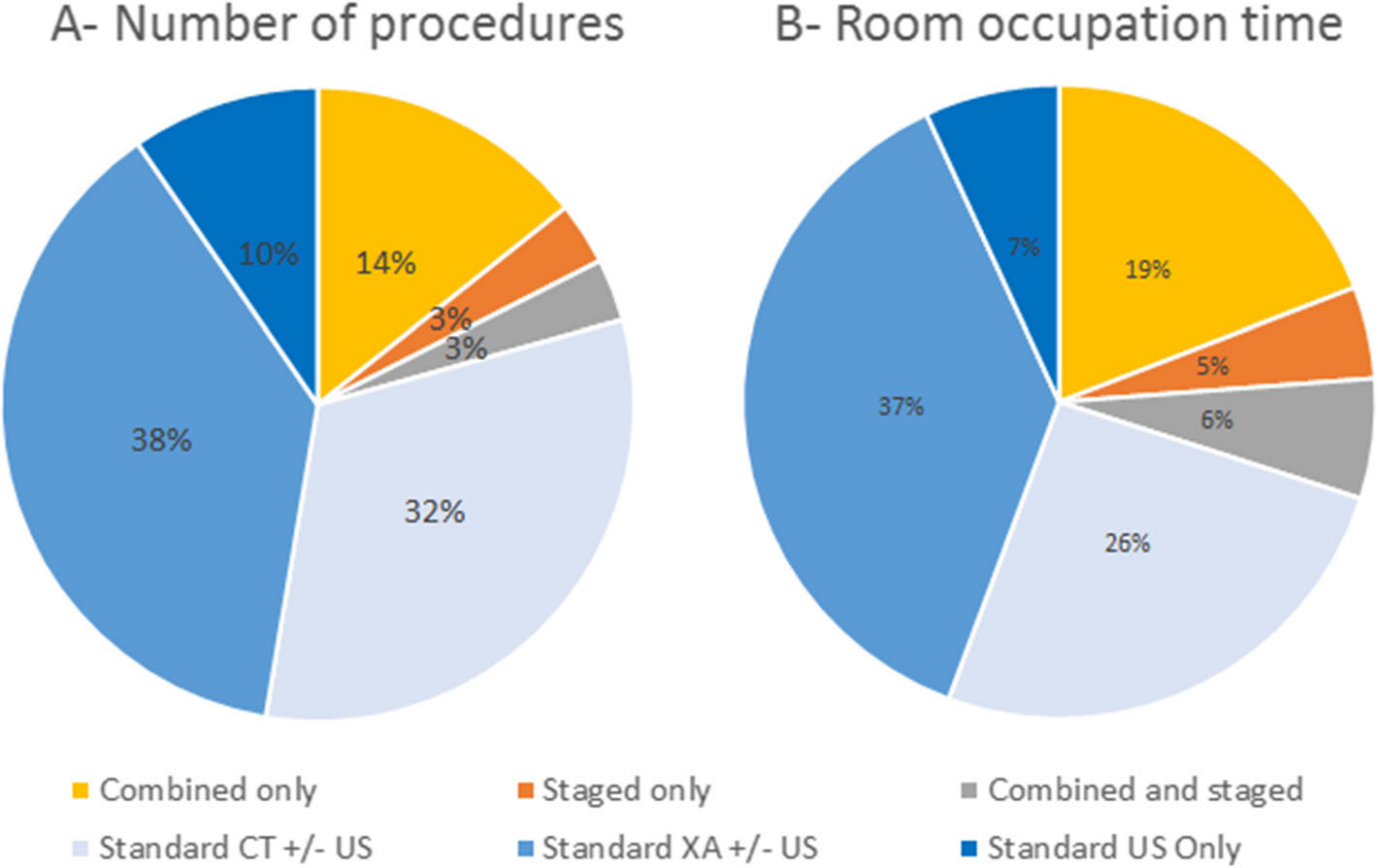
Contribution of each procedure type to the overall number of procedures (**a**) and total room occupation time (**b**)

The average procedure time in the ACT suite was 1 h and 48 min. The ACT suites occupation time were allocated at 70% to Standard procedures, 19% to Combined procedures, 5% to Staged, and 6% to a combination of both (Fig. 1).

#### Standard procedures

The ACT rooms were primarily used for Standard procedures both in terms of number of procedures and occupation time. Among the Standard procedures, fluoroscopic guidance was the most frequently used in ACT suites, representing 38% of the volume of procedures and 37% of total occupation time, followed by the CT guidance with 32% of the volume of procedures and 26% in terms of occupation time. US only procedures represented 7% of the ACT occupation time for 10% of the volume of procedures.

In terms of procedure types, biopsies and drainages represents more than half of Standard procedures (54%) performed in ACT suites; procedures occurrences and room occupation time are shown in Table 2.

**Table 2:** Standard procedures occurrences and average room occupation time

Procedure description	N	Percent	Room occupation minutes (mean +/−SD)	
Drainage, body	444	15.6%	85	+/− 44
Biopsy, body	329	11.6%	80	+/− 44
Biopsy, spine	273	9.6%	94	+/− 34
Biopsy, chest	257	9.0%	76	+/− 37
Biliary intervention, stent or catheter placement or conversion to stent	225	7.9%	112	+/− 48
Venous intervention	204	7.2%	132	+/− 80
Venous access device	202	7.1%	79	+/− 30
GU intervention	181	6.4%	88	+/− 42
Drainage, chest	166	5.8%	83	+/− 41
Arterial intervention (non-liver embolization, arteriogram, stent, pump angio)	82	2.9%	175	+/− 75
Biopsy, bone	69	2.4%	84	+/− 33
Spine intervention (non-biopsy/aspiration)	61	2.1%	146	+/− 47
Biopsy, neuro non-spine	52	1.8%	83	+/− 25
Enteral tube placement/exchange	49	1.7%	106	+/− 52
Other	46	1.6%	82	+/− 25
US Doppler extremity lower vein bilateral	32	1.1%	74	+/− 57
Ablation, chest	29	1.0%	133	+/− 45
SIRT mapping/treatment	24	0.8%	125	+/− 44
Ablation, body	18	0.6%	173	+/− 64
Ablation, soft tissue	11	0.4%	198	+/− 67
Ablation, bone non-spine	10	0.4%	131	+/− 43
Lymphatic intervention	10	0.4%	220	+/− 44
Liver embolization	6	0.2%	212	+/− 42
Others	68	2.4%		
Total	2872		96	+/− 53

An average Standard procedure took less than 2 h (96 +/− 53 min), with the fluoroscopy-only procedures averaging the longest time (107 +/− 60 min) while US-only procedures (78 +/− 44 min) averaged the shortest time, just below CT-only procedures (88 +/− 44 min).

#### Combined procedures

The Combined procedures using the full capability of the ACT suite (including the ones that were also classified as Staged) represented 17% of the volume of procedures and 25% of the room occupation time. Combined procedures lasted 2.5 h (145 +/− 70 min) on average whereas average Combined and Staged procedures lasted more than 3.5 h (210 +/− 83 min).

**Fig. 2:**
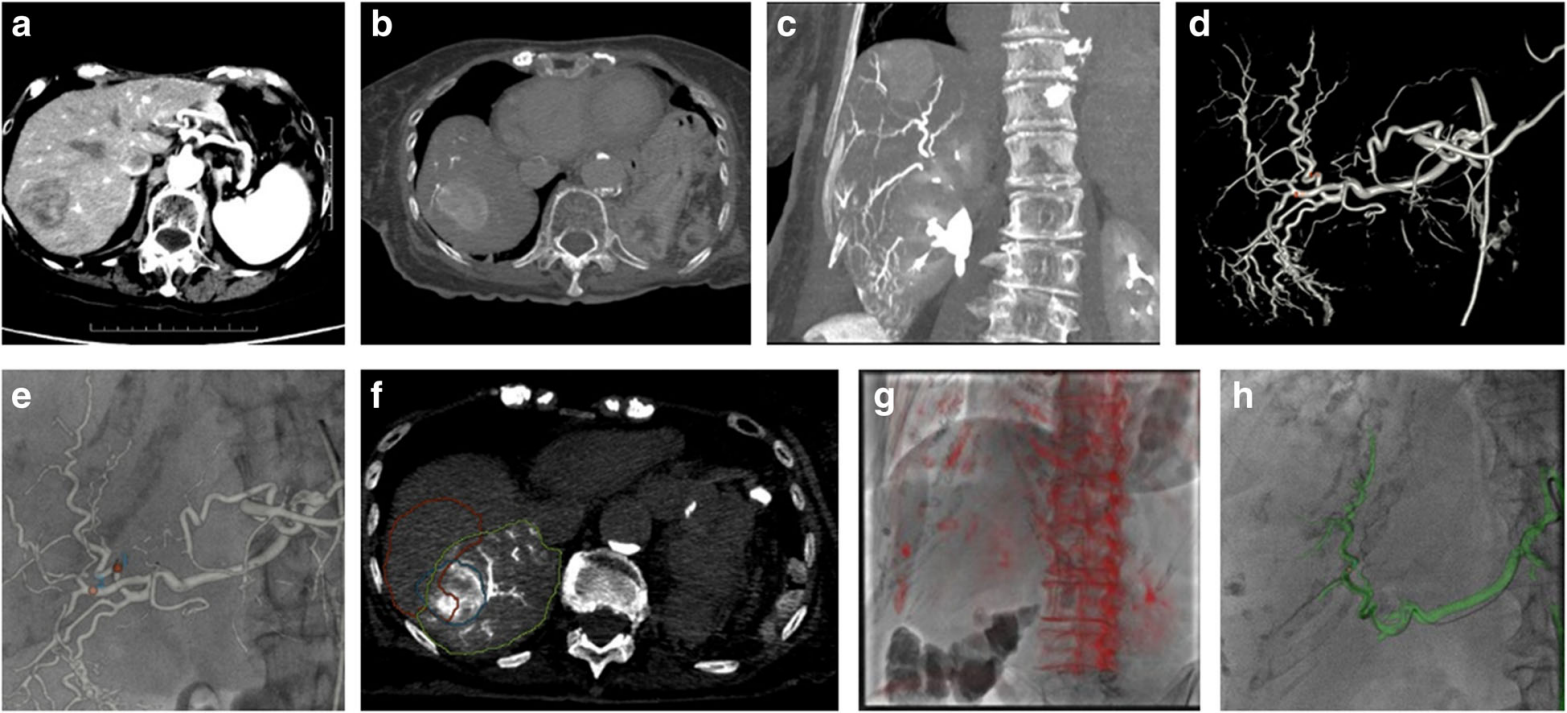
Example of CT usage during a Y90 procedure: The patient was a 90 year old female with a history of high-grade leiomyosarcoma of the gastric wall resected 3 years ago. A heterogeneous mass of 4.0 × 3.2 cm appeared in segment 7 on follow-up imaging (**a**) and the patient was referred to interventional radiology for SIRT Y90 treatment. A catheter- injected CT was performed from the proper hepatic artery, and anatomy was reviewed in reformatted views (**b**, **c**). A 3D model of the vessels was automatically generated and vessels were marked with potential MAA injection points (**d**, arrows), which were then overlaid on the fluoroscopy for guidance (**e**). An additional catheter-injected CT was performed from the desired location, which was then used to compute the tumor and perfused parenchyma volumes (**f**). The day of the Y90 treatment, registration of the same dataset using bony landmarks (**g**) was initially performed and the desired vessel was displayed for guidance (**h**)

**Fig. 3:**
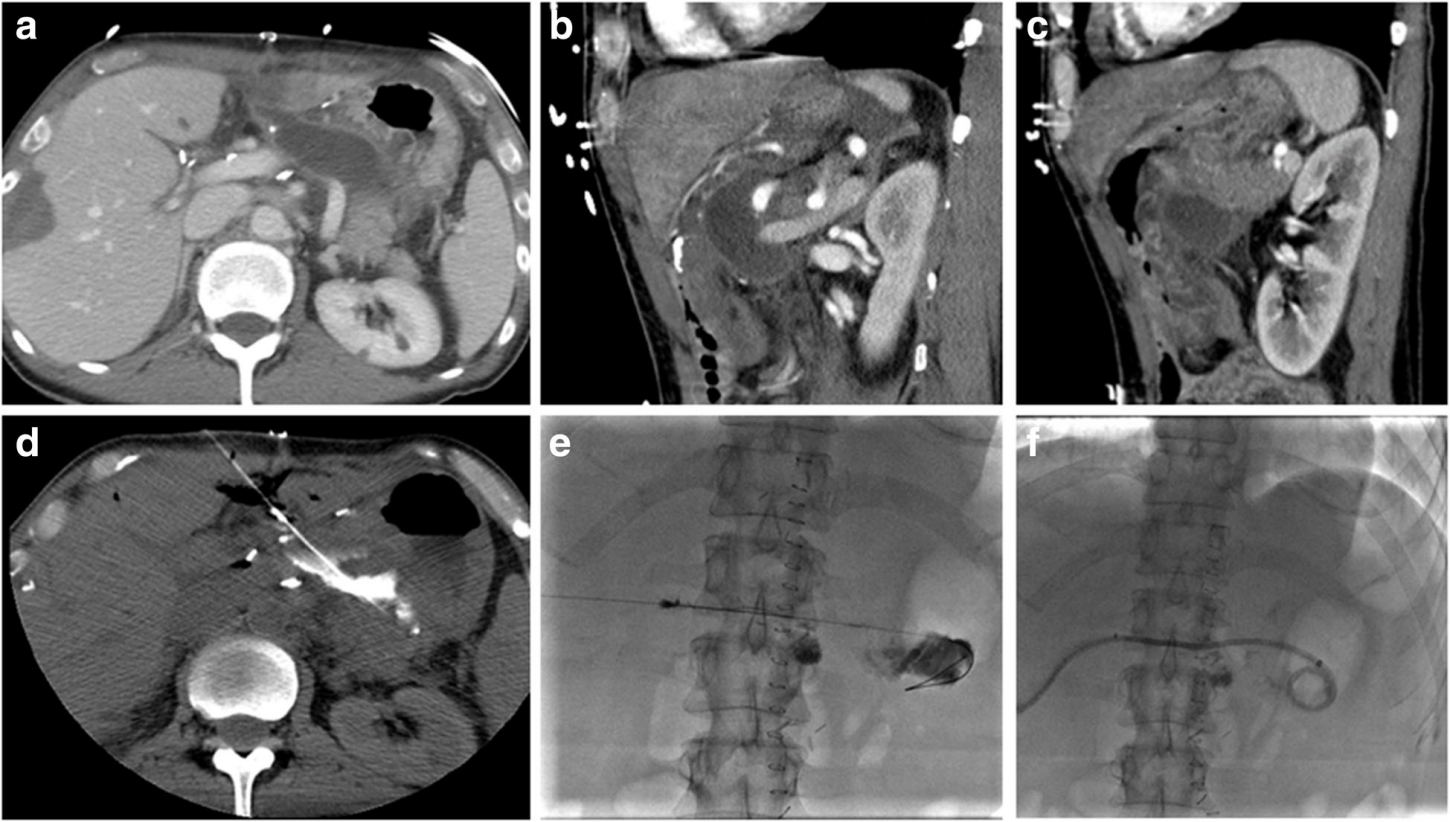
Example of CT usage during a drainage procedure: The patient was a 59 year old female with a postoperative bleed near the pancreatico-gastric anastomosis following liver wedge resection (**a**). Access from anterior and lateral approaches was not possible because of the left liver (**b**) and stomach locations (**c**). Posterior approach was difficult due to the left kidney and splenic arteries. CT (**d**) was very useful to guide the needle track in 3 dimensions. Once the target had been reached, the operator switched from CT to angiography (**e**) to set up the drain (**f**)

Table 3 describes the most frequently performed Combined procedures and the reason for use of both modalities. The main usage of the CT when combined with a fluoroscopy-guided procedure was for immediate contrast retention assessment after hepatic tumor bland embolization, catheter-injected CT at a particular location to assess the anatomy of interest and guide the procedure in 3D (Fig. 2), or drain location confirmation (Fig. 3). For Combined procedures with a percutaneous access, CT fluoroscopy was used before transitioning to fluoroscopy guidance or assessment in two cases: for difficult accesses and for temporarily displacing organs in situations where some temporary organ shift was necessary to preserve organ health prior to a treatment such as ablation or radiation therapy.

**Table 3:** Combined procedures

Procedure description	N	Percent	Room occupation minutes (mean +/− SD)		Primary reason for angio	Primary reason for CT
Drainage, body	170	33%	95	+/− 38	Drain formation check	Abscess access
Liver embolization	132	26%	179	+/− 61	Catheter guidance	Post-embolization non-injected CT
SIRT mapping/treatment	90	18%	194	+/−64	Catheter guidance	Catheter injected CT for tumor delineation, arterial mapping, extra-hepatic blush identification
Arterial intervention (non-liver embolization, arteriogram, stent, pump angio)	27	5%	187	+/−70	Catheter guidance	Catheter injected CT for tumor delineation, arterial mapping, extra-hepatic blush identification
Drainage, chest	26	5%	104	+/− 51	Drain formation check	Abscess access
Biliary intervention, stent or catheter placement or conversion to stent	25	5%	143	+/− 77		
Enteral tube placement/exchange	16	3%	125	+/−98		
GU intervention, stent or catheter placement/conversion	8	1%	116	+/−43		
Lymphangiogram +/− thoracic duct embolization	6	1%	225	+/− 65		
Others	14	3%				
Total	514		145	+/− 70

#### Staged procedures

The Staged procedures (including the ones that were also classified Combined) represented 6% of the total procedure volume and 11% of the room occupation time, with an average occupation time of 2.5 h (159 +/− 64 min). For Staged procedures, the different imaging modalities were used to guide 2 successive interventions merged in 1 procedure (details in Table 4). In most of the Staged interventions, CT was used for percutaneous intervention (biopsies, ablation, drainage) and fluoroscopy for vascular navigation.

**Table 4:** Occurrences and average room occupation time for Staged procedures (including Combined and Staged Procedures)

Procedure description	N	Percent	Room occupation minutes (mean +/−SD)	
Multiple collection drains	43	20%	141	+/− − 55
Liver embolization + ablation	26	12%	237	+/− 73
Liver embolization + biopsy	25	12%	221	+/− 56
Kyphoplasty + ablation + biopsy	19	9%	176	+/− 27
Venous access + biopsy	15	7%	126	+/− 37
Kyphoplasty + biopsy	10	5%	142	+/− 56
Biopsy + collection drain	9	4%	150	+/− 84
GU intervention + biopsy	9	4%	133	+/− 48
GU intervention + collection drainage	9	4%	136	+/− 58
Biliary intervention + biopsy	8	3%	143	+/− 35
Embolization + biopsy + ablation	5	2%	303	+/− 67
Others	39	18%		
Total	217		159	+/− 64

### Staged ACT procedure vs equivalent single modality room procedures: liver tumor embolization and ablation

Multiple drain collections and liver tumor embolization followed by ablation were the most common staged procedures. Whereas the need for CT guidance during biopsies or drainages is subjective, CT guidance for liver tumor ablation is a widespread practice.

**Fig. 4:**
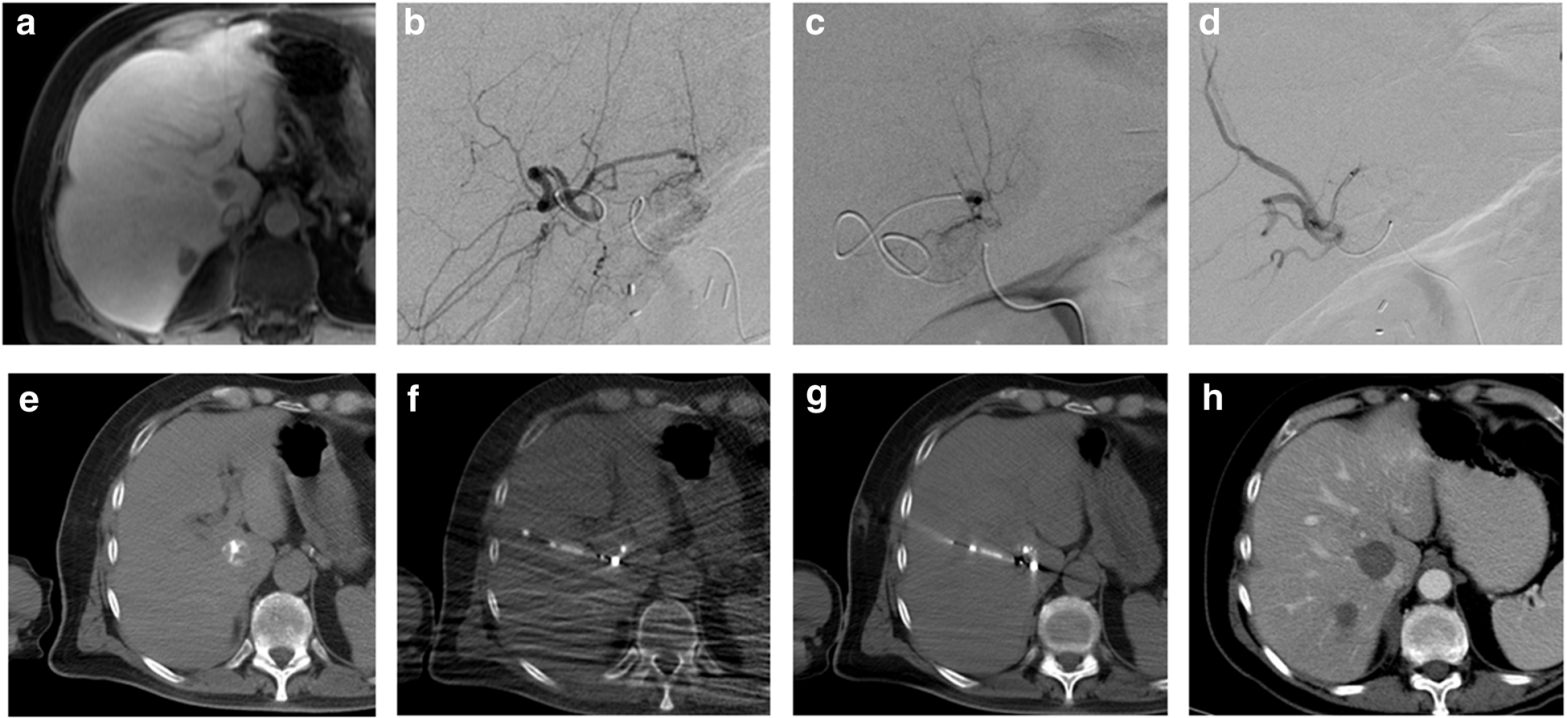
Example of liver ablation and embolization performed in an ACT room: The patient was a 61 year old male with 2 liver adrenal cortical carcinoma metastases in segment 7 (**a**) shown by the pre-operative contrast-enhanced MR (**a**). Selective bland embolization was performed under fluoroscopic guidance (**b**, **c**, **d**). The embolic agent facilitated tumor identification on the CT (**e**) and contributed to the guidance of the ablation probe under CT fluoroscopy (**f**). The probe treatment position was confirmed by a last CT before ablation (**g**). CT follow-up after 5 days show satisfying ablation margins (**h**)

From January 2016 to September 2019, 84 Staged procedures for liver tumor embolization and ablation were performed in the ACT suites. The tumor embolization was performed under fluoroscopic guidance in a first step in most cases, as the embolic agent was then visible on the CT and could help to localize the tumor for the ablation. The CT was then used for ablation probe guidance and margins assessment (Fig. 4).

Over the same period, 2 equivalent procedures were evaluated in single modality suites: liver tumor ablation and liver tumor embolization. The room occupation time in the Staged fashion was significantly shorter (*p* < 0.01) with an average of 217 min versus 300 min of cumulative average room time for single ablation (141 min) and single embolization (159 min). More surprisingly, the average procedure time was also significantly shorter (p < 0.01) in the Staged fashion at 159 min instead of 178 cumulative minutes for ablation (74 min) and embolization (104 min) performed separately.

## Discussion

As can be seen from the results, there are many potential advantages of an ACT room over regular CT and IR rooms, which are relevant to the patient, the treating team, and the scheduling team. One of the most important advantages is the flexibility that the system offers for case scheduling and management. In a regular setting, where single modality rooms are being used, cases are scheduled upfront into specific rooms based on the imaging modality usually used in similar cases; this scheduling challenge is made irrelevant in an ACT room which can accommodate any intervention. In this study only 20% of the procedures used the full capacity of the room, which suggests that actually the flexibility of the room for scheduling purposes may actually be the most important benefit at this institution. These scheduling advantages are often overlooked, with prior authors mostly discussing procedure related advantages and disadvantages of the ACT room over the standard angiography room [2].

Cone Beam CT acquisition on angiography systems has improved over the recent years and is gaining popularity as it allows for high quality 3D imaging and advanced processing in the IR room. This enables the performance of complex procedures in a single modality room. Nevertheless, as pointed out by Bapts et al., this acquisition mode still suffers from limitations [13] such as limited 3D reconstruction field of view, limited contrast resolution, slower spin rates, and higher sensitivity to various artifacts compared to CT imaging. For complex procedures where the team thinks there is high risk for complications or difficult percutaneous access requiring cross sectional imaging, the ACT room provides greater imaging comfort and remains an appropriate solution for the treating teams. Such procedures were a minority in this study but are important scenarios to take into consideration in everyday practice. ACT was also useful in cases where more than one body cavity was involved. The ability to quickly assess large body volume and to use both CT and fluoroscopy interchangeably was very valuable in the setting of multiple fluid collection which may have involved the pleural, abdominal, and pelvic cavities altogether.

Our results showed that the full capacities of ACT suites were used in 20% of cases representing 30% of room occupation time. The operator used both fluoroscopy and CT guidance in 17% of cases, meaning that those complex procedures required both modalities and might be difficult to perform in regular IR settings. It was also shown that certain procedures were performed quicker when performed in a Staged fashion in an ACT room versus separately in 2 sessions. ACT room utilization could be maximized by performing more of these Staged procedures instead of doing 2 Standard procedures on the same patient one after the other. Combination of biopsies with other interventions, such as venous access device placement, tumor embolization, or ablation are some of the best candidate procedures for this purpose. Combining such procedures in the same intervention has benefits from the patient perspective: a shorter duration usually means less anesthesia or sedation time, as well as shorter in-patient stay or fewer out-patient visits to the hospital.

An ACT room can be useful in big and specialized hospitals where flexibility of the room for complex cases and related scheduling issues is needed, but even in small, non-specialized hospitals, an ACT room can offer a good solution compared to a combination of a regular CT room and fluoroscopy suite in a limited space.

One limitation of this study is that it was based on a single cancer center’s experience with a specific patient population and highly oncologic-oriented physicians, which prompts unique usage of the ACT room. In addition, the configuration of the ACT room facilitates the use of CT over cone beam CT whenever cross-sectional imaging is needed. For some of the procedures, CT might be used instead of cone beam CT for logistical reasons; therefore these procedures were considered Staged or Combined in this study though there was no specific need for CT. A common example was when a standard liver embolization became a combined procedure due to the use of non-contrast CT at the end of the procedure instead of a standard cone beam CT. Finally, both the financial and radiation dose aspects of the ACT rooms versus standard IR suite utilization were not covered in this study; future studies will be performed to address these issues.

## Conclusions

This study showed that the ACT system has a role in the current and future interventional oncology field, with advantages for both the patient and the treating team. Further thought and experience is needed to optimize the proper utilization of the ACT room.

## Data Availability

The datasets used and/or analyzed during the current study are available from the corresponding author on reasonable request.

## Abbreviations

ACT: Angiography Computed Tomography
CT: Computed Tomography
US: Ultrasound Scanner
